# Real-world treatment patterns and clinical outcomes of patients with primary biliary cholangitis in the United States

**DOI:** 10.57264/cer-2025-0172

**Published:** 2026-08-12

**Authors:** Nisreen Shamseddine, Hongbo Yang, Su Zhang, Dongni Ye, Shravanthi Seshasayee, Jingyi Chen, Sonal Kumar, Kris V Kowdley

**Affiliations:** 1 Ipsen, Cambridge, MA 02142, US; 2 Analysis Group, Inc., Boston, MA 02199, US; 3 Weill Cornell Medical College, Cornell University, New York, NY 10065, US; 4 Liver Institute Northwest, Seattle, WA 98105, US

**Keywords:** fenofibrate, gemfibrozil, obeticholic acid, primary biliary cholangitis, ursodeoxycholic acid

## Abstract

**Background & aim:**

Primary biliary cholangitis (PBC) is a chronic cholestatic liver disease that can lead to increased morbidity and mortality. This study described real-world treatment patterns and clinical outcomes by line of treatment among patients with PBC in the US.

**Materials & methods:**

Adults (≥18 years) diagnosed with PBC on or after 1 January 17 were identified in the IQVIA PharMetrics^®^ Plus database and grouped into newly diagnosed, first-line (1L) and second-line or more (2L+) cohorts. Index date was initial PBC diagnosis or initiation of 1L or 2L therapy; follow-up continued until the earliest of end of continuous enrollment, death or data end. Time to treatment initiation, treatment discontinuation and negative clinical outcomes were assessed with Kaplan–Meier analysis.

**Results:**

The newly diagnosed, 1L and 2L+ cohorts included 1748, 1659 and 181 patients, respectively (average age at index: 52.7–54.5 years; female: 84.2–89.0%). Of the newly diagnosed cohort, 34.8% did not initiate PBC treatment within 1.5 years post-diagnosis. In the 1L cohort, median time from diagnosis to 1L initiation was 1.2 months; median time from 1L initiation to 1L discontinuation/2L initiation was nearly 5 years. In the 2L+ cohort, median time from 2L initiation to 2L discontinuation was approximately 4 years. In the untreated, 1L, and 2L+ cohorts, 18.9%, 14.4% and 19.3% of patients developed ≥1 negative clinical outcome post-index (usually cirrhosis).

**Conclusion:**

Results of this US population-based study demonstrate a potential unmet need for early intervention and effective treatment options for patients with PBC, as one in three patients with PBC remain untreated years after diagnosis.

## Plain language summary: How primary biliary cholangitis is treated in clinical practice in the US and patient outcomes

**What is this article about?:**

Primary biliary cholangitis (PBC) is a rare liver disease that gradually worsens over time and can be potentially life-threatening. People with PBC may try multiple medications to manage their disease and/or treat their symptoms because the first one does not always work.

**What methodology is described?:**

In this article, we describe a study that looked at how patients with PBC were treated with medication in the US and what the results of these treatments were.

**What were the results?:**

The majority (65.2%) of patients with newly diagnosed PBC started at least one medication for PBC. Almost half (48.0%) of patients with newly diagnosed PBC started a medication within 1 month of diagnosis and nearly half of patients stopped their first medication within 4 years of starting it. About one in three newly diagnosed patients did not start any PBC medication by one and a half years after their diagnosis. Additionally, about one in five patients had a negative health complication, regardless of the treatment they started. Usually, this meant their disease got worse and led to severe liver scarring, known as cirrhosis.

**What do the results mean?:**

This study shows that patients with PBC may not be receiving timely or effective treatment with medications for PBC. More awareness of PBC and its treatment options is important to help connect patients with the right care at the right time.

Primary biliary cholangitis (PBC) is a chronic cholestatic liver disease that results in progressive damage to the small intrahepatic bile ducts, predominantly affecting women aged 40–60 years [1]. The estimated prevalence of PBC is approximately 39.2 per 100,000 people in the US as of 2014, although estimates have varied depending on the population assessed [2,3]. Patients with PBC are at risk of disease progression to chronic cholestasis, cirrhosis and end-stage liver disease [4,5]. While some are without obvious symptoms, a substantial proportion of patients with PBC experience fatigue (25–76%) [6,7], abdominal pain (17–33%) [8] and pruritus (29–69%) [6,7,9,10]. Accordingly, PBC can have a detrimental impact on a patient’s quality of life and daily functioning, even in early disease stages [11,12]. The prognosis of the disease is primarily based on the likelihood of cirrhosis development and subsequent liver failure, which can shorten a patient’s lifespan [13].

There are limited treatment options for patients with PBC in the US. Until June 2024, there were only two pharmacological therapies approved by the US Food and Drug Administration (FDA): ursodeoxycholic acid (UDCA) and obeticholic acid (OCA) [14,15]. The most recent (2021) American Association for the Study of Liver Diseases treatment guidelines recommend UDCA for first-line (1L) treatment of PBC until the patient experiences an inadequate response or toxicity, while OCA is currently recommended as a second-line (2L) treatment after UDCA or for patients who are intolerant of UDCA as of September 2025 [5,16]. However, in November 2024, the Food & Drug Administration (FDA) recommended against full approval of OCA and did not find substantial evidence of its clinical benefit; OCA was voluntarily withdrawn from the US market following a request by the FDA in 2025 [17,18]. Only a proportion of patients with PBC respond to UDCA and OCA – up to 40% of patients receiving UDCA as 1L treatment and 50% receiving OCA as 2L treatment fail to meet PBC OCA International Study of Efficacy (POISE) response criteria; in addition, a small (3–5%) proportion of patients who receive UDCA are unable to tolerate the treatment [19–21]. Due to their known effects on cholesterol and bile acid homeostasis [22], fibrates have also been used for patients with incomplete response to UDCA; however, their use is off-label in the US [5]. The US FDA has since approved other 2L treatments: the two proliferator-activated receptor agonists elafibranor and seladelpar (approved in the US in June 2024 and August 2024, respectively) [23–25]. Linerixibat, an ileal bile acid transporter, was approved in March 2026, specifically for the treatment of cholestatic pruritus [26]. However, at the time of this analysis, the only 2L therapy available in the US for the treatment of PBC was OCA, and fibrates were used off-label [5,16].

Lack of treatment or delayed treatment is reported to be associated with worse PBC prognosis and contributes to increased morbidity, mortality and medical resource use [3,13,27–29]. However, few studies have examined treatment patterns and clinical outcomes of patients with PBC in the real world, and none in recent years. In order to address this knowledge gap, the current study aimed to describe the real-world treatment patterns and clinical outcomes among patients with PBC in the US by line of treatment from 2016 to 2022, including the time to treatment initiation, treatment discontinuation and negative clinical outcomes.

## Materials & methods

### Data source, sample identification and cohort creation

This observational retrospective cohort study used the IQVIA PharMetrics^®^ Plus database (January 2016–December 2022), which contains fully adjudicated and integrated medical and pharmacy claims data for more than 210 million beneficiaries in the US since 2006. This database did not include lab testing results, and subsequently treatment response and certain PBC prognosis factors (i.e., alkaline phosphatase and total bilirubin levels) could not be directly assessed in the present study. As this study used de-identified claims data, no ethical review was required.

**Figure 1.**
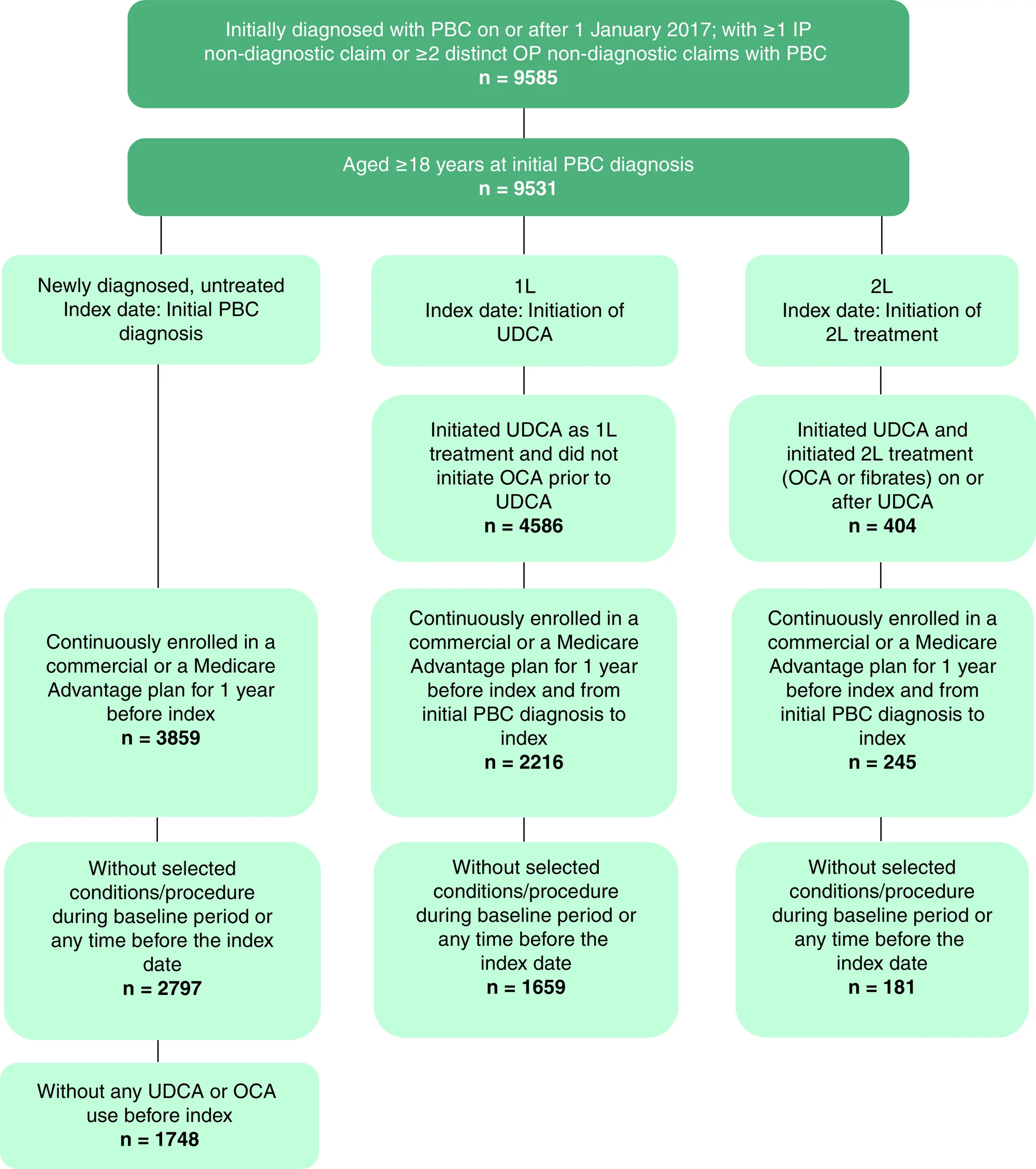
Patient selection flow chart. ^a^Selected conditions/procedure during baseline period or any time before index include liver cirrhosis, hepatic decompensation, clinically significant portal hypertension, primary sclerosing cholangitis and hepatocellular carcinoma during baseline period and liver transplant any time before the index date. 1L: First-line; 2L+: Second-line or more; IP: Inpatient; OCA: Obeticholic acid; OP: Outpatient; PBC: Primary biliary cholangitis; UDCA: Ursodeoxycholic acid.

Adults (aged ≥18 years) with an initial diagnosis of PBC on or after 1 January 2017, were identified in the database based on *International Classification of Disease, 9th revision, Clinical Modification* (ICD-9-CM) code 571.6 or ICD-10-CM code K743. Three nonmutually exclusive cohorts were created by line of treatment: a newly diagnosed cohort, including patients with newly diagnosed, untreated PBC; a 1L cohort, including patients who initiated UDCA as the 1L treatment; and a 2L+ cohort, including patients who initiated a 2L treatment (i.e., OCA or fibrates [i.e., fenofibrate and gemfibrozil]). Across cohorts, patients were required to be continuously enrolled in a commercial or Medicare Advantage plan for ≥1 year before index and from initial PBC diagnosis to index (if applicable). Patients were also required to be without the following conditions and procedures during the baseline period or any time before index: liver cirrhosis (defined as compensated cirrhosis), hepatic decompensation (defined as a diagnosis of any of the following complications/conditions: unspecified jaundice, ascites, end-stage liver disease, hepatic encephalopathy, hepatorenal syndrome, and bleeding esophageal varices), clinically significant portal hypertension (as there are no diagnosis codes specific to clinically significant portal hypertension, this was defined by proxy as a diagnosis of portal hypertension with splenomegaly or thrombocytopenia/low platelet count or a diagnosis of portal hypertension with any varices), primary sclerosing cholangitis (PSC; patients with PSC were identified as patients with a diagnosis of PSC in addition to a diagnosis of ulcerative colitis, Crohn’s disease or inflammatory bowel disease to preclude the misdiagnosis of PBC as PSC), hepatocellular carcinoma (HCC) and liver transplant. Diagnosis and procedure codes used to identify the above conditions and procedures, respectively, can be found in Supplementary Table 1, and a cohort selection flowchart with detailed sample selection criteria is presented in Figure 1.

**Figure 2.**
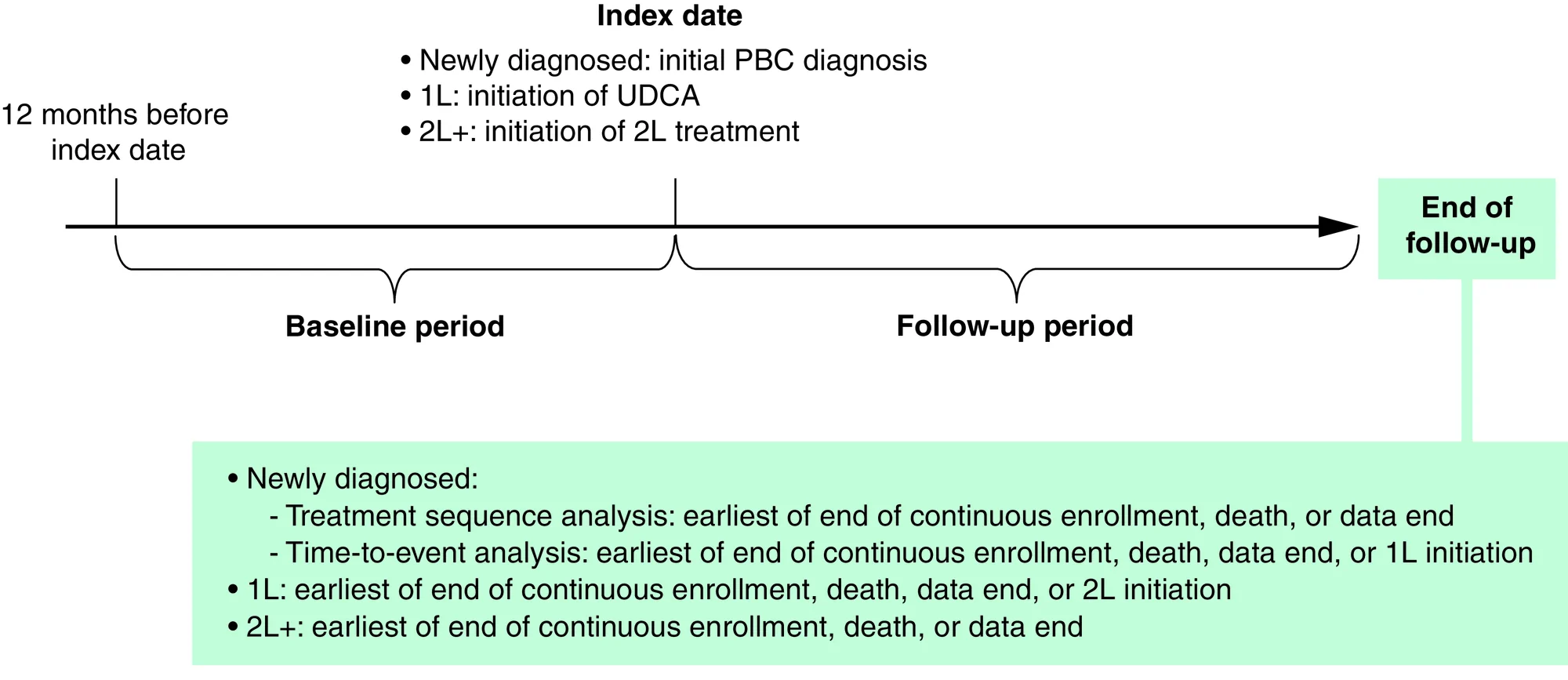
Index date, baseline period, and follow-up period for each cohort. 1L: First-line; 2L+: Second-line or more; PBC: Primary biliary cholangitis; UDCA: Ursodeoxycholic acid.

The baseline period was the 12 months prior to the index date, defined as the initial PBC diagnosis for the newly diagnosed cohort, the initiation of UDCA for the 1L cohort and the initiation of 2L treatment for the 2L+ cohort. For the treatment sequence analysis (conducted in the newly diagnosed cohort), the end of the follow-up period was the earliest of the end of continuous enrollment, death or data end. For the clinical outcomes analyses (conducted across all cohorts), the end of the follow-up period was the earliest of the end of continuous enrollment, death or data end for all cohorts; for the newly diagnosed and 1L cohorts, the follow-up period also ended at the time of 1L or 2L initiation, respectively, as described in Figure 2.

### Study outcomes

The study outcomes included patient characteristics and comorbidities during the baseline period, treatment patterns following PBC diagnosis, time to treatment initiation and discontinuation and time to negative clinical outcomes during the follow-up period. Comorbidity burden was described with the Charlson Comorbidity Index (CCI); comorbid liver diseases and complications (autoimmune hepatitis, liver fibrosis and portal hypertension); and other disorders common among patients with PBC (i.e., dyslipidemia, hypertension, Type 2 diabetes, etc.). Negative clinical outcomes included liver transplantation, liver cirrhosis, inpatient hospitalization for hepatic decompensation, clinically significant portal hypertension, HCC and all-cause death. See above for definitions for these negative clinical outcomes; diagnosis codes can be found in Supplementary Table 1.

Time to treatment initiation/discontinuation included the time from the index date to the initiation of 1L treatment for the newly diagnosed cohort; the time from the index date to the discontinuation of 1L treatment or the initiation of 2L treatment, whichever was earlier, for the 1L cohort; and the time from index date to discontinuation of 2L treatment for the 2L+ cohort. Treatment initiation was defined as the first prescription claim date of the respective PBC treatment after the index date, while treatment discontinuation was defined as a gap of at least 30 days between the end of treatment supply and the end of the follow-up period (i.e., the earliest of end of continuous enrollment, death or end of data period [i.e., December 2022]). Patients were censored at the end of follow-up.

### Statistical analysis

Baseline characteristics and comorbidities were summarized descriptively for each cohort as counts and proportions or means with standard deviations (SD). For patients in the newly diagnosed cohort, baseline characteristics were also separately summarized for those who did not initiate any PBC treatment after the initial PBC diagnosis during the entire follow-up period.

Post-index treatment patterns were described for each study cohort. The observed follow-up duration for each cohort was summarized descriptively using median and interquartile range (IQR). The treatment sequence, up to two lines of therapy after the initial PBC diagnosis, was described for the newly diagnosed cohort using a Sankey diagram. The time from index to treatment initiation (i.e., time from the initial diagnosis to 1L initiation for the newly diagnosed cohort, as well as 1L initiation to 2L initiation for the 1L cohort), time to treatment discontinuation, time to the earliest negative clinical outcome (as listed in the above section), and time to each individual negative clinical outcome were described for each cohort using Kaplan–Meier analysis. For the newly diagnosed cohort, the time to earliest negative clinical outcome and time to each individual negative clinical outcome analysis were assessed in patients who did not initiate any PBC treatment after initial PBC diagnosis only. Analyses were conducted using R (version 3.6.2).

## Results

### Baseline characteristics

Among 9585 patients who were initially diagnosed with PBC after 1 January 2017, 1748, 1659 and 181 were identified in the database, met study criteria and were included in the newly diagnosed cohort, 1L cohort, and 2L+ cohort, respectively (Figure 1). Among the newly diagnosed cohort, 609 patients (34.8%) did not initiate any PBC treatment after initial PBC diagnosis (i.e., untreated) while the remaining 1139 patients in the cohort initiated ≥1 PBC treatment. Although not all patients in the newly diagnosed cohort initiated PBC treatment, a higher rate of untreated patients in the newly diagnosed cohort had specialist gastroenterologist visits during follow-up (63.1%, n = 384) than patients in the newly diagnosed cohort who initiated treatment (37.1%, n = 423). The median (IQR) follow-up time for the treatment sequence analysis in the newly diagnosed cohort was 19.6 (9.2, 36.5) months.

The baseline demographic and clinical characteristics are presented in Table 1. Across the three main cohorts, patients were, on average, 52.7–54.5 years old at index; 84.2–89.0% were female; and the majority (93.3–95.0%) were covered by a commercial plan. Baseline comorbidity burden was the highest in the 2L+ cohort (mean CCI [SD]: 2.5 [0.9]), followed by the 1L (2.1 [1.4]) and the newly diagnosed (1.5 [1.6]) cohorts. For liver complications, more patients in the 2L+ cohort had autoimmune hepatitis (16.0%) at baseline than in the newly diagnosed (6.9%) and 1L (12.1%) cohorts. Additionally, portal hypertension was observed in the newly diagnosed (0.6%) and 1L (0.7%) cohorts, but not in the 2L+ cohort. Dyslipidemia (43.9–54.7%) and hypertension (41.2–43.6%) were the most prevalent comorbidities in each cohort, and symptoms such as fatigue (19.9–24.4%) were also prevalent in all cohorts. The prevalence of baseline Type 2 diabetes was highest in the 2L+ cohort (19.3% vs 16.2% and 15.1% in the newly diagnosed and 1L cohort, respectively); similarly, pruritus was highest in the 2L+ cohort (18.8% vs 5.4% and 7.5%).

**Table 1.** Baseline characteristics.

	Newly diagnosed (N = 1748)	Newly diagnosed, did not initiate PBC treatment, untreated (N = 609)	1L (N = 1659)	2L+ (N = 181)
Demographic characteristics				
Age at index (years), mean ± SD	53.9 ± 11.3	52.9 ± 12.9	54.5 ± 10.4	52.7 ± 10.1
Female, n (%)	1471 (84.2%)	470 (77.2%)	1465 (88.3%)	161 (89.0%)
Insurance plan				
Commercial	1632 (93.4%)	566 (92.9%)	1548 (93.3%)	172 (95.0%)
Medicare Advantage	116 (6.6%)	43 (7.1%)	111 (6.7%)	9 (5.0%)
**Clinical characteristics**				
CCI, mean ± SD	1.5 ± 1.6	1.4 ± 1.7	2.1 ± 1.4	2.5 ± 0.9
**Comorbid liver complications, n (%)**				
Autoimmune hepatitis	121 (6.9%)	30 (4.9%)	200 (12.1%)	29 (16.0%)
Liver fibrosis	47 (2.7%)	7 (1.1%)	83 (5.0%)	8 (4.4%)
Portal hypertension	10 (0.6%)	3 (0.5%)	12 (0.7%)	0 (0.0%)
**Other comorbidities, n (%)**				
Dyslipidemia	767 (43.9%)	225 (36.9%)	806 (48.6%)	99 (54.7%)
Hypertension	720 (41.2%)	246 (40.4%)	707 (42.6%)	79 (43.6%)
Type 2 diabetes	283 (16.2%)	104 (17.1%)	251 (15.1%)	35 (19.3%)
Urinary tract infection	174 (10.0%)	65 (10.7%)	167 (10.1%)	16 (8.8%)
Gallstone disease	169 (9.7%)	66 (10.8%)	190 (11.5%)	14 (7.7%)
Osteoporosis	96 (5.5%)	33 (5.4%)	112 (6.8%)	19 (10.5%)
Raynaud’s syndrome	54 (3.1%)	22 (3.6%)	58 (3.5%)	4 (2.2%)
Autoimmune thyroid disease	49 (2.8%)	13 (2.1%)	61 (3.7%)	8 (4.4%)
Sjögren’s syndrome	47 (2.7%)	9 (1.5%)	61 (3.7%)	11 (6.1%)
Systemic lupus erythematosus	43 (2.5%)	16 (2.6%)	48 (2.9%)	7 (3.9%)
Ulcerative colitis	43 (2.5%)	21 (3.4%)	33 (2.0%)	2 (1.1%)
Crohn’s disease	27 (1.5%)	13 (2.1%)	24 (1.4%)	1 (0.6%)
**Symptoms, n (%)**				
Fatigue	403 (23.1%)	148 (24.3%)	404 (24.4%)	36 (19.9%)
Pruritus	94 (5.4%)	31 (5.1%)	125 (7.5%)	34 (18.8%)
Diarrhea	157 (9.0%)	65 (10.7%)	141 (8.5%)	14 (7.7%)

1L: First line; 2L+: Second line or more; CCI: Charlson Comorbidity Index; PBC: Primary biliary cholangitis; SD: Standard deviation.

Untreated patients had lower rates of dyslipidemia (36.9%) and autoimmune hepatitis (4.9%) than patients in the overall newly diagnosed, 1L and 2L+ cohorts (Table 1). The other baseline characteristics of this sub-cohort were generally comparable with those of the overall newly diagnosed cohort.

### Treatment sequence

**Figure 3.**
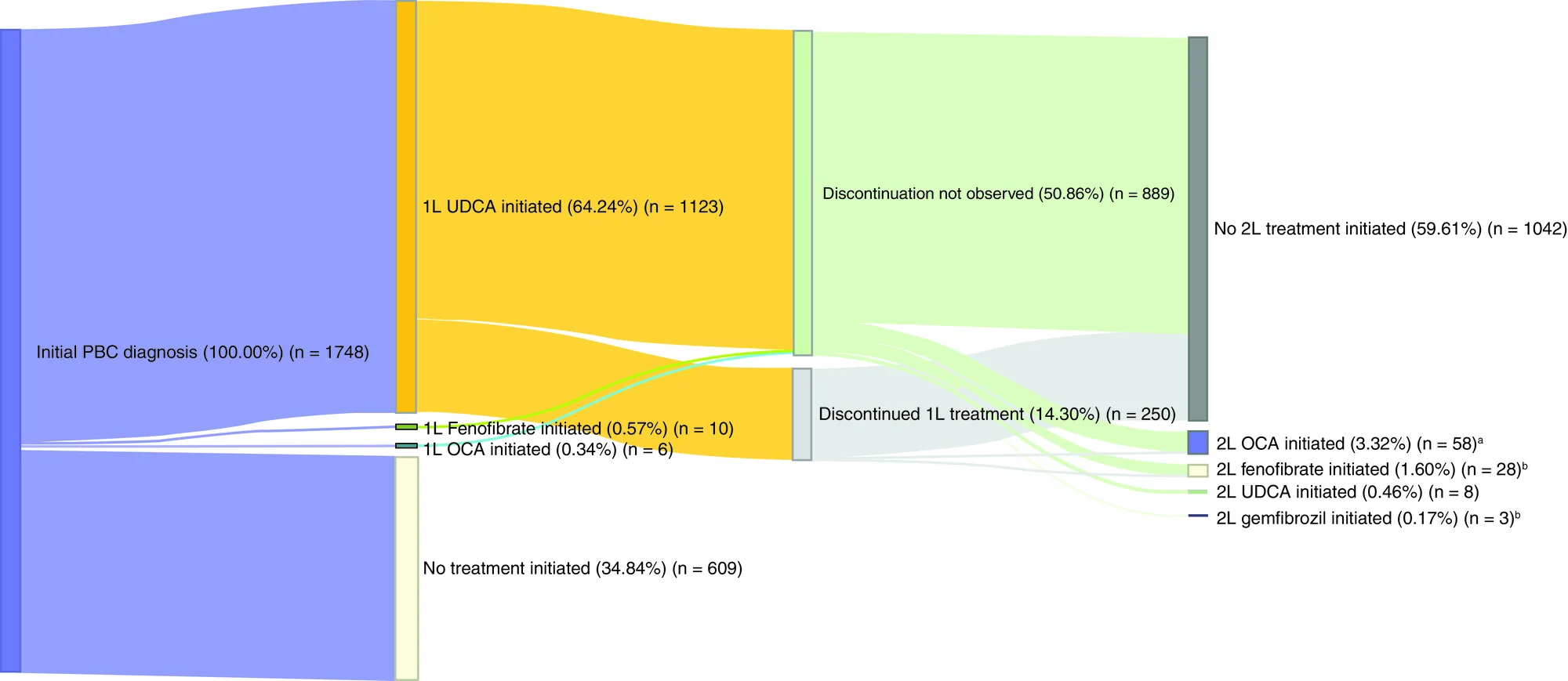
Distribution of treatment sequences in the newly diagnosed cohort. ^a^Among 58 patients who initiated 2L OCA, 3 patients discontinued UDCA prior to OCA initiation, and 55 patients did not. ^b^Among 31 patients who initiated 2L fibrates, 3 patients discontinued UDCA prior to initiation of fibrates, and 28 patients did not. 1L: First-line; 2L: Second-line; OCA: Obeticholic acid; PBC: Primary biliary cholangitis; UDCA: Ursodeoxycholic acid.

In the newly diagnosed cohort, 1139 (65.2%) patients initiated ≥1 PBC treatment, primarily UDCA as the 1L treatment (98.6%, n = 1123) (Figure 3). Of those who initiated 1L treatment, 250 patients discontinued 1L treatment and 244 of these patients did not subsequently initiate 2L treatment. In total, 97 patients who initiated 1L treatment subsequently initiated 2L treatment, with more than half (56.7%, n = 55) using OCA + UDCA as the 2L treatment.

### Time to treatment initiation & time to treatment discontinuation

**Figure 4.**
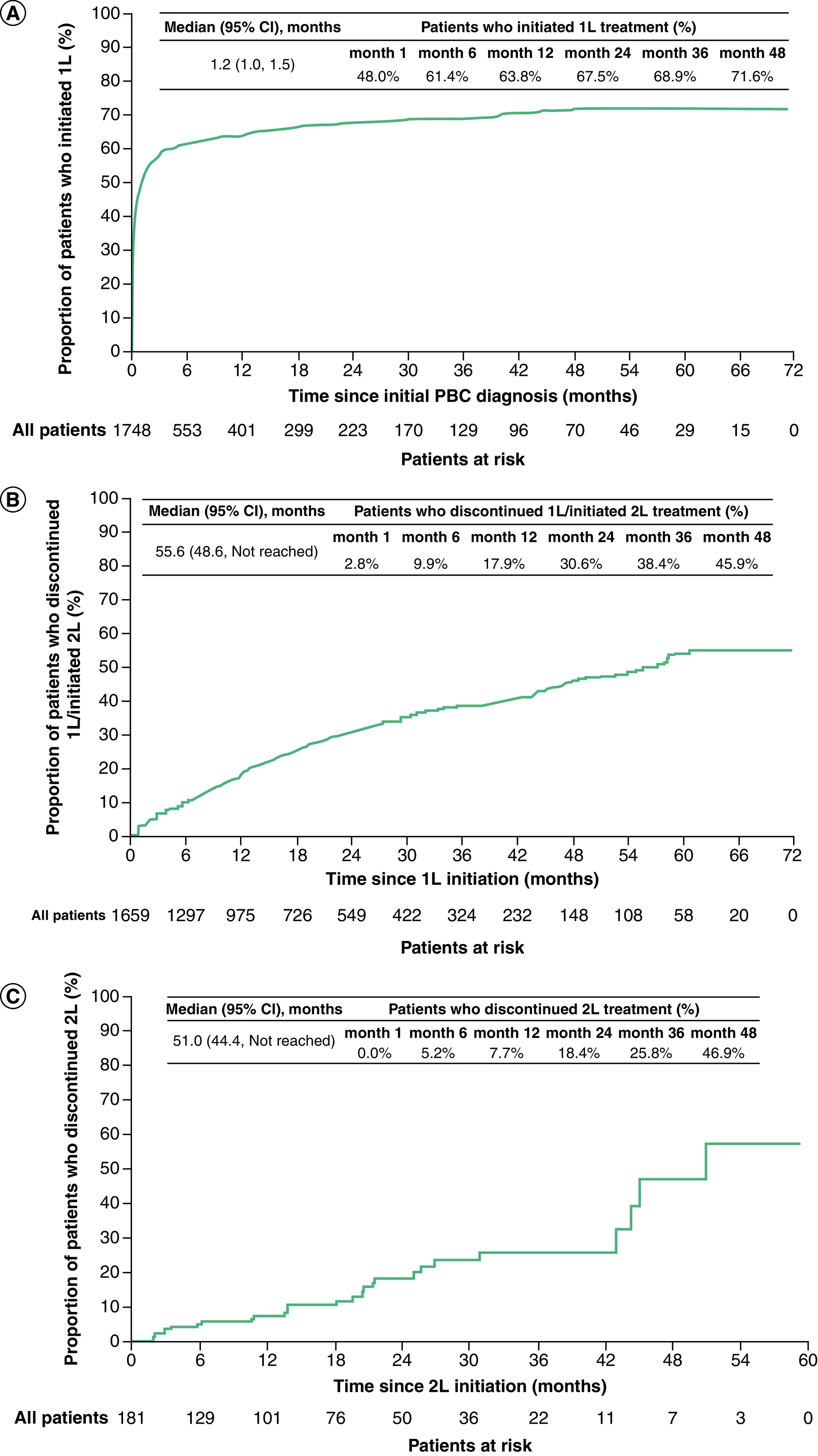
Time to 1L and 2L treatment initiation and discontinuation. **(A)** Time to 1L initiation in the newly diagnosed cohort. **(B)** Time to 1L discontinuation/2L initiation in the 1L cohort. **(C)** Time to 2L discontinuation in the 2L+ cohort. Time to **(A)** 1L initiation in the newly diagnosed cohort, **(B)** 1L discontinuation/2L initiation in the 1L cohort and **(C)** 2L discontinuation in the 2L+ cohort are shown in this figure. 1L: First-line; 2L+: Second-line or more; PBC: Primary biliary cholangitis.

In the newly diagnosed cohort, nearly half (48.0%) of patients initiated treatment within 1 month of PBC diagnosis, with a median time from diagnosis to 1L initiation of 1.2 months. The cumulative incidence of 1L treatment initiation by 6 and 48 months post-index was 61.4% and 71.6%, respectively (Figure 4A). In the 1L cohort (i.e., those who initiated UDCA as 1L treatment and were separately selected for inclusion in the 1L cohort; see Figure 1 for detailed selection criteria), a total of 484 patients discontinued 1L treatment or initiated 2L treatment (2L monotherapy or combination treatment with 1L). The median time from 1L initiation to 1L discontinuation/2L initiation was 55.6 months. The estimated cumulative incidence of patients who discontinued 1L treatment or initiated 2L treatment by 12 months of follow-up was 17.9% and 45.9% by 48 months of follow-up (Figure 4B). While not presented in the figure, among the 1659 patients in the 1L cohort, 352 patients discontinued 1L UDCA without/before 2L treatment initiation; 12 of these 352 patients initiated 2L treatment after UDCA discontinuation, while the remaining 340 patients did not initiate 2L treatment by the end of follow-up after 1L UDCA discontinuation. Additionally, among the 144 (8.7%) patients in the 1L cohort who initiated 2L treatment during follow-up, 132 added OCA/fibrates (including 88 patients who added OCA and 44 patients who added fibrates) to existing UDCA as the 2L treatment, while the remaining 12 patients discontinued UDCA before initiating OCA/fibrates. The median time from 1L treatment discontinuation to 2L treatment initiation was 3.1 months. The estimated cumulative incidence of patients in the 1L cohort who initiated 2L treatment by 12 and 48 months of follow-up was 4.3%, and 14.0%, respectively. In the 2L+ cohort (i.e., those who initiated 2L treatment and were separately selected), the median time from 2L initiation to 2L discontinuation was 51.0 months. Nearly half of the patients had discontinued 2L treatment 4 years later (48 months post 2L initiation) (Figure 4C).

### Time to first negative clinical outcomes

#### Untreated cohort

For clinical outcomes analyses, the median (IQR) duration of the follow-up period was 1.1 (0.07, 10.9) months for the newly diagnosed cohort. Within the newly diagnosed cohort, the median (IQR) duration of follow-up was 15.0 (6.7, 31.0) months for patients who remained untreated and 5.0 (1.0, 34.5) days for patients who initiated any PBC treatment (the end of follow-up for the clinical outcomes analyses in the newly diagnosed cohort was the earliest of end of continuous enrollment, death, data end or 1L initiation, as described in Figure 2). Thus, patients who started treatment after initial diagnosis could be included in the newly diagnosed, 1L and 2L+ cohort analyses of negative clinical outcomes, if 1L and 2L treatments were initiated after diagnosis, respectively.

**Figure 5.**
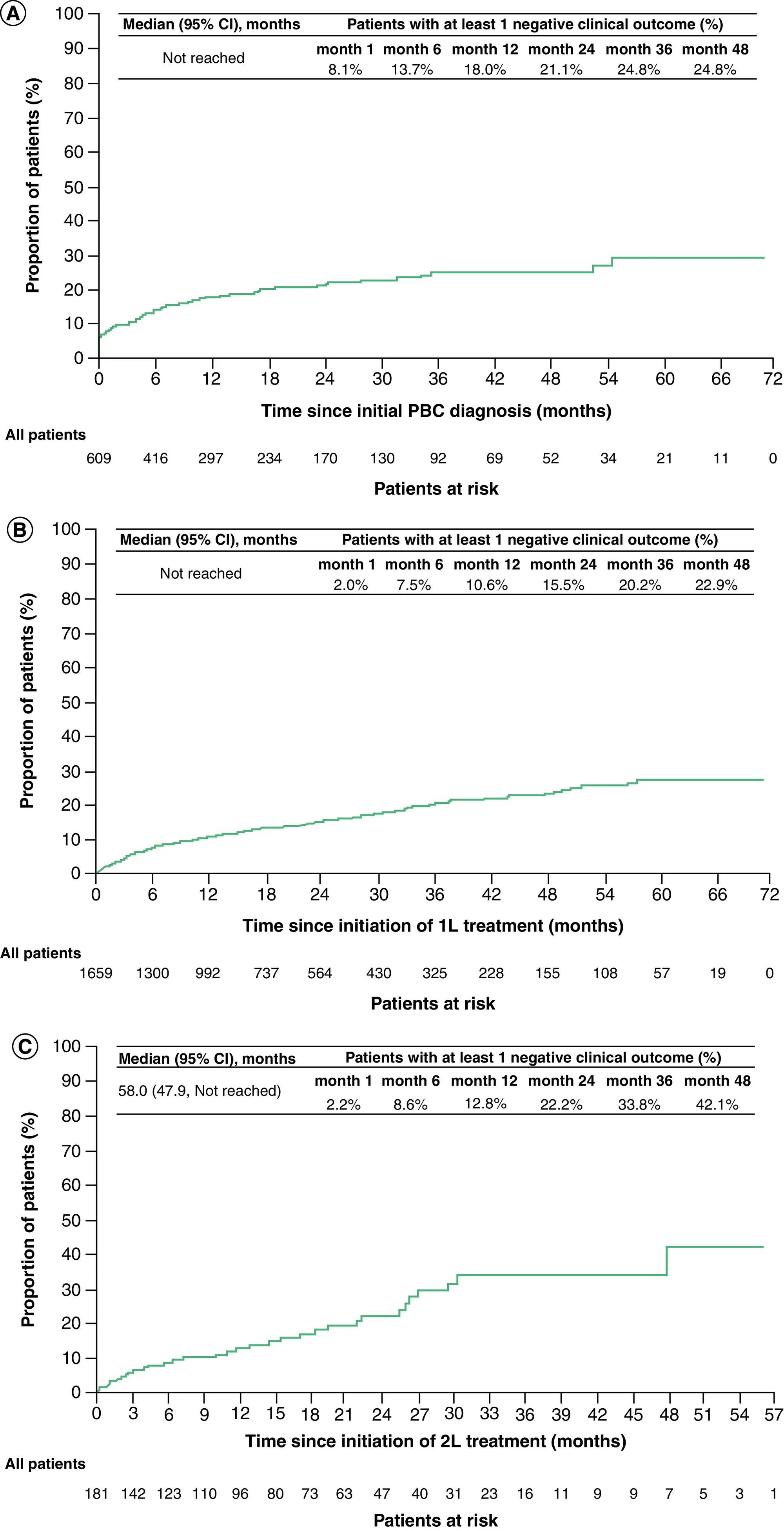
Time to the earliest negative clinical outcome. **(A)** Untreated cohort. **(B)** 1L cohort. **(C)** 2L+ cohort. This figure assesses the time to the earliest negative clinical outcome in the **(A)** newly diagnosed, untreated cohort, **(B)** 1L cohort and **(C)** 2L+ cohort. 1L: First-line; 2L+: Second-line or more; PBC: Primary biliary cholangitis.

Among untreated patients (n = 609), 115 (18.9%) developed at least 1 negative clinical outcome, with liver cirrhosis being the most common first negative clinical outcome (60.0%, n = 69). Over 20% of patients developed at least 1 negative clinical outcome within 2 years (24 months) of diagnosis (Figure 5A). In the analyses of the time to individual negative clinical outcomes, 16% of patients had liver cirrhosis and nearly 10% of patients underwent hospitalization for hepatic decompensation by 24 months, respectively (Supplementary Figure 1).

#### 1L cohort

In the 1L cohort, over a median (IQR) follow-up duration of 18.5 (9.2, 35.0) months since the start of 1L treatment, 239 (14.4%) patients developed at least 1 negative clinical outcome; liver cirrhosis was the most common first negative clinical outcome (88.7%, n = 212). Approximately 16% of patients developed at least 1 negative clinical outcome within 24 months (Figure 5B). In the analyses of the time to individual negative clinical outcomes, 14% of patients had liver cirrhosis and nearly 2% were hospitalized for hepatic decompensation by 24 months (Supplementary Figure 2).

#### 2L+ cohort

In the 2L+ cohort, over a median (IQR) follow-up duration of 15.2 (5.8, 29.7) months since the start of 2L treatment, 35 (19.3%) patients developed at least 1 negative clinical outcome. Similar to the other cohorts, liver cirrhosis was the most common first negative clinical outcome (88.6%, n = 31). A total of 22% of patients developed at least 1 negative clinical outcome within 24 months (Figure 5C). In the analyses of the time to individual negative clinical outcomes, 22% of patients had liver cirrhosis and 4% underwent hospitalization for hepatic decompensation by 24 months (Supplementary Figure 3). By the end of the follow-up, no patients in this cohort had received a liver transplant or died.

## Discussion

This retrospective observational study is among the first to assess the real-world treatment patterns and clinical outcomes by line of treatment in patients diagnosed with PBC in the US. The results indicated that approximately one-third (35%) of patients with PBC did not initiate therapy upon diagnosis, even after a median follow-up of 1.5 years. Over one in five patients (20%) in the untreated cohort developed at least 1 negative clinical outcome during the follow-up period, typically cirrhosis. Consistent with US treatment guidelines for PBC that recommend UDCA as a first-line therapy until patients experience inadequate response or toxicity [5], UDCA was the predominant 1L therapy. However, among the newly diagnosed patients who initiated 1L UDCA and later discontinued it, 59.6% did not initiate any 2L treatment, placing them at risk of disease progression.

A higher treatment rate was reported in an observational, cross-sectional study by MacDonald *et al.* based on patients with PBC treated in a single urban health system with a liver transplant program [30]. Among 495 patients included in the study, 471 (95%) were treated with UDCA ± OCA/fibrates. Our research differs from this prior study in that it longitudinally follows up with a more general PBC population. Indeed, a more comparable treatment rate has been reported in studies based on similar study designs and patient populations. For example, a real-world study by Lu *et al.* using electronic health records of approximately 4200 US patients with PBC (2003–2014) from the fibrotic liver disease (FOLD) consortium health systems, demonstrated that 70% were treated with UDCA over a median follow-up of 5 years [31]. This estimate may be slightly higher than that in our study due to the longer follow-up period in FOLD.

Patients with PBC were observed to have multiple comorbidities at baseline, and the burden generally escalated by line of therapy, with the highest prevalence of most of the disorders among the 2L+ cohort. Across all cohorts, the most prevalent comorbidities were dyslipidemia and hypertension, affecting nearly half of patients, while 19.9–24.4% reported fatigue and 5.1–18.8% reported pruritus. These symptoms may be under-reported, as real-world data have shown that patients with PBC often lack documentation of self-reported fatigue and pruritus in their medical records [32,33]. Compared with those who initiated treatment, newly diagnosed patients with PBC who did not initiate a 1L treatment (i.e., untreated) were slightly younger, had a reduced rate of certain liver complications (i.e., autoimmune hepatitis and liver fibrosis), and a lower proportion were female. A higher proportion of untreated patients were observed to develop at least one negative clinical outcome during follow-up compared with the 1L cohort (18.9% vs 14.4%). However, in the absence of laboratory data or measures of disease severity at baseline, it is not possible to definitively determine whether these differences reflect underlying variations in disease severity or other factors at baseline. As a result, difference in outcomes may reflect underlying patient or disease characteristics rather than treatment effects.

Overall, at least one in five patients across cohorts developed at least 1 negative clinical outcome associated with PBC during follow-up, per the estimated cumulative incidence rates, before initiating treatment (i.e., untreated patients) or switching treatments (i.e., the initiation of 2L treatment for the 1L cohort). Across all cohorts, liver cirrhosis was the most common first and overall negative clinical outcome (one in five to one in six patients across cohorts), while hospitalization for hepatic decompensation was the second most common negative clinical outcome (1 in 50 to 1 in 10 patients across cohorts). Similar findings have been reported in existing literature assessing the real-world burden of PBC in the US, as liver cirrhosis is a major and well-established complication of PBC [4,34]. For example, a study using Nationwide Inpatient Sample data (2005–2014) found cirrhosis to be present in 37% of patients with PBC [35]. The avoidance or delay of cirrhosis is a major treatment goal as patients with liver cirrhosis may have a worse prognosis and shortened lifespan than those without the condition [13,36].

Hepatic decompensation is another common complication of PBC, observed among 23.7% of patients with PBC in a prospective study in China [37], and the incidence of decompensation events can escalate over time [38]. Incomplete UDCA response or inconsistent UDCA treatment are both risk factors for hepatic decompensation among patients with PBC [37], underscoring the importance of effective, lifelong therapy to slow the rate of disease progression.

Although disease severity could not be determined and the follow-up data were variable, the observed treatment patterns and burden of outcomes in the current study point to a potential unmet need for patients with PBC in the US. The strengths of this study include the timeliness of the data, the large database with rich, real-world data in the US, and the ability to examine treatment patterns by line of therapy. While randomized controlled trials provide strong internal validation of hypotheses, studies using real-world data provide external validation against a more representative population than found in clinical trials [39]. However, administrative claims-based studies are subject to selection bias as they primarily include individuals who seek medical care and have insurance, potentially excluding uninsured or untreated populations [40]. This study is also subject to other general limitations of analyses using administrative claims data, including the potential for incorrectly documented diagnosis codes and the inability to capture medical services or pharmacy dispensing obtained outside of a patient's plan, such as a detailed patient medical history. Incorrectly documented diagnosis codes may be an issue particularly for diagnoses of cirrhosis and PBC, which was formerly known as primary biliary cirrhosis; subsequently, chart validation of these diagnoses may be most accurate [41]. Additionally, most beneficiaries in the PharMetrics Plus data are covered by commercial plans; therefore, the results from this study may not to be generalizable to patients with PBC covered by other healthcare plans, such as Medicare and Medicaid. Furthermore, lab testing results were not available in the PharMetrics Plus data used in this study. As such, some important prognosis factors for PBC that are routinely measured to assess treatment response, such as the levels of alkaline phosphatase and total bilirubin, cannot be evaluated in the current study. In addition, treatment discontinuation was not directly observed in this study. Instead, treatment discontinuation was defined as a gap of at least 30 days between the end of treatment supply and end of follow-up period. This approach may have resulted in overestimation of treatment discontinuation, as some gaps could reflect nonadherence, delayed refills or other factors rather than true treatment cessation. While unlikely, it is also possible that treatment with UDCA was not always captured, either due to the timing of the treatment (i.e., treatment occurred prior to registration in the PharMetrics Plus database) or miscoding. Finally, patients with negative clinical outcomes assessed during baseline were excluded from the study; therefore, the true prevalence of these outcomes in this patient population is likely to be underestimated. Without the inclusion of baseline laboratory data or measures of disease severity, it is not possible to determine a causal relationship to treatment effects, and the outcomes may reflect underlying patient or disease characteristics. Future research should utilize databases providing lab testing data to investigate the underlying reasons for treatment assignment, such as the timing of treatment, and to evaluate responses to these interventions. Additionally, the current analysis does not fully reflect the impact of the more recently approved 2L PBC therapies, elafibranor and seladelpar. As the treatment landscape continues to evolve, further research is needed to assess whether their use influences 2L treatment initiation and mitigates the burden of PBC observed in this study.

## Conclusion

This retrospective US population-based study found that approximately one-third of patients with PBC did not receive treatment within 1.5 years of initial PBC diagnosis. Among patients who did initiate 1L treatment, up to 1 in 5 patients developed at least 1 PBC-related negative clinical outcome within 4 years after initial diagnosis/treatment initiation, with liver cirrhosis being the most common negative clinical outcome to develop first. Approximately 3 in 5 newly diagnosed patients who initiated 1L treatment did not initiate any 2L treatment, further highlighting the importance of appropriate and prompt assessment of 1L treatment response to identify patients at risk for disease progression or patients who may benefit from additional therapies [5,12]. Taken together, these findings highlight the potential unmet medical need of patients with PBC in the US, particularly increasing awareness of the disease course and treatment options, including the timely initiation of safe and effective therapy for PBC.

## Summary points

- Primary biliary cholangitis (PBC) is a chronic cholestatic liver disease that results in progressive damage to the small intrahepatic bile ducts.
- Timely and appropriate treatment is important to mitigate the clinical burden of PBC, which can worsen over time with lack of or delayed treatment.
- Few studies have examined treatment patterns or clinical outcomes in the real world and this study addresses this knowledge gap by examining the real-world treatment patterns and clinical outcomes by line of treatment in adult patients with PBC in the United States, using claims data from the the IQVIA PharMetrics^®^ Plus database (January 2016–December 2022).
- In this study, three nonmutually exclusive cohorts were created by line of treatment: a newly diagnosed cohort (n = 1748), including patients with newly diagnosed, untreated PBC; a first-line (1L) cohort (n = 1659), including patients who initiated ursodeoxycholic acid (UDCA) as the 1L treatment; and a 2L+ cohort (n = 609), including patients who initiated a second-line (2L) treatment (i.e., OCALIVA^®^ or fibrates [i.e., fenofibrate and gemfibrozil]).
- Despite the availability of treatment, approximately one-third of patients with PBC did not receive treatment within 1.5 years of initial PBC diagnosis.
- In the newly diagnosed cohort, 65.2% of patients initiated ≥1 PBC treatment, primarily UDCA as the 1L treatment (98.6%). Of those who initiated 1L UDCA and later discontinued it, 59.6% did not initiate any 2L treatment, placing them at risk of disease progression.
- In the 1L cohort, median time from diagnosis to 1L initiation was 1.2 months; median time from 1L initiation to 1L discontinuation/2L initiation was nearly 5 years. In the 2L+ cohort, median time from 2L initiation to 2L discontinuation was approximately 4 years.
- In the untreated, 1L, and 2L+ cohorts, 18.9%, 14.4% and 19.3% of patients developed ≥1 negative clinical outcome post-index (usually liver cirrhosis).
- Overall, the findings of this study demonstrate the need for increased awareness among healthcare providers of the PBC disease course and treatment options, which in turn may benefit patients through improvements in patient care.

## Supplementary Material


