# Supplementary material for: Real-world treatment patterns and clinical outcomes of patients with primary biliary cholangitis in the United States
Source: J Comp Eff Res. 2026 Aug 12;15(9):e250172. doi: 10.57264/cer-2025-0172 (PMC13556886; doi:10.57264/cer-2025-0172)
Supplement: Supplementary file 1 [file cer-15-250172-s1.docx]

# SUPPLEMENTARY MATERIALS

# TABLE

**Supplementary Table 1. Diagnosis and procedure codes used in the eligibility criteria, baseline characteristics, and clinical outcomes analyses.**

| **Medical conditions** | **ICD-10-CM codes** | |
| --- | --- | --- |
| **Conditions involved in the eligibility criteria and clinical outcomes analyses** | | |
| Liver cirrhosis | K74.6x (other and unspecified cirrhosis of the liver) | |
| Hepatic decompensation | R17 (unspecified jaundice)  R18.0, R18.8 (ascites)  K72.0x, K72.1x, K72.9x (end-stage liver disease)  K76.82 (hepatic encephalopathy)  K76.7 (hepatorenal syndrome)  I85.01, I85.11 (bleeding esophageal varices) | |
| Clinically significant portal hypertension^a^ | K76.6 (portal hypertension)  R16.1, R16.2, D73.81, D73.2 (splenomegaly)  D69.6, D69.49, D69.59, D69.3 (thrombocytopenia/low platelet count)  I85.x (esophageal varices)  I86.4 (gastric varices) | |
| Hepatocellular carcinoma | C22.0 | |
| Primary sclerosing cholangitis^b^ | K83.01 | |
| Crohn’s disease | K50.xx | |
| Ulcerative colitis | K51.xx | |
| Inflammatory bowel disease | K52.x | |
| **Other conditions summarized in the baseline characteristics** | | |
| Autoimmune hepatitis | K75.4 | |
| Liver fibrosis | K74.00–K74.02 (hepatic fibrosis)  K74.1 (hepatic sclerosis)  K74.2 (hepatic fibrosis with hepatic sclerosis) | |
| Dyslipidemia | E78.0–E78.5 | |
| Hypertension | I10 (essential [primary] hypertension) I11 (hypertensive heart disease)  I12 (hypertensive chronic kidney disease)  I13 (hypertensive heart and chronic kidney disease)  I15 (secondary hypertension) I16 (hypertensive crisis) | |
| Type 2 diabetes | E11.xx | |
| Urinary tract infection | N39.0 | |
| Gallstone disease | K80.xx | |
| Osteoporosis | M80.0x, M80.8x, M81.0, M81.6, M81.8 | |
| Raynaud’s syndrome | I73.00–I73.01 | |
| Autoimmune thyroid disease | E06.3 | |
| Sjögren’s disease | M35.0x | |
| Systemic lupus erythematosus | M32.x | |
| Fatigue | R53.82 (chronic fatigue, unspecified)  R53.83 (other fatigue) | |
| Pruritus | L29.x | |
| Diarrhea | R19.7 (diarrhea, unspecified)  K59.1 (functional diarrhea) | |
| **Procedures** | **CPT codes** | **ICD-10-PCS codes** |
| Liver transplant | 47135 (liver allotransplantation) | 0FY00Z0 (allogenic)  0FY00Z1 (syngeneic)  0FY00Z2 (zooplastic) |

**Abbreviations:** CPT, Current Procedural Terminology; ICD-10-CM, International Classification of Diseases, Tenth Revision, Clinical Modification; ICD-10-PCS, International Classification of Diseases, Tenth Revision, Procedure Coding System; PBC, Primary biliary cholangitis; PSC, Primary sclerosing cholangitis.

^a^Clinically significant portal hypertension was defined by proxy as a diagnosis of portal hypertension with splenomegaly or thrombocytopenia/low platelet count or a diagnosis of portal hypertension with any varices; ^b^Patients with PSC were identified as patients with a diagnosis of PSC in addition to a diagnosis of ulcerative colitis, Crohn’s disease, or inflammatory bowel disease to preclude the misdiagnosis of PBC as PSC.

# FIGURES

**Supplementary Figure 1. Time to each individual clinical outcome in the untreated cohort.**

**Abbreviations:** HCC, Hepatocellular carcinoma; IP, Inpatient; PBC, Primary biliary cholangitis.

Supplementary Figure 2. Time to each individual clinical outcome in the 1L cohort.

**Abbreviations:** 1L, First-line; HCC, Hepatocellular carcinoma; IP, Inpatient.

Supplementary Figure 3. Time to each individual clinical outcome in the 2L+ cohort.

**Abbreviations:** 2L+, Second-line or more; HCC, Hepatocellular carcinoma; IP, Inpatient.
